# The state of global palliative care research: a bibliometric study

**DOI:** 10.3332/ecancer.2025.1943

**Published:** 2025-07-10

**Authors:** Mevhibe B Hocaoglu, Grant Lewison, Hamish Sharp, Tania Pastrana, Eve Namisango, James Cleary, Barbara Hasties, Eric Kabisa, Helena Musau, Kathryn Spangenberg, Paola Ruiz, Zipporah Ali, Mertixell Mallafre-Larrosa, Alfredo Polo, Julie Torode, Ajay Aggarwal, Richard Sullivan

**Affiliations:** 1 Cicely Saunders Institute, King’s College London, London, UK; 2 King’s College London, Institute of Cancer Policy, Guy’s Hospital, London SE1 9RT, UK; 3 Department of Palliative Medicine, Medical Faculty RWTH Aachen University, Aachen, Germany; 4 African Palliative Care Association, Kampala, Uganda; 5 Supportive Oncology, Department of Medicine, Division of Hematology/Oncology, Indiana University School of Medicine, Indianapolis, IN, USA; 6 Team Humanity International, Amsterdam, The Netherlands; 7 Rwanda Palliative Care and Hospice Organisation (RPCHO), Kigali, Rwanda; 8 Kenyatta University Teaching Research and Referral Hospital, Nairobi, Kenya; 9 Komfo Anokye Teaching Hospital, Kumasi, Ghana; 10 La Asociación Cuidados Paliativos de Colombia ASOCUPAC, Bogotá, Colombia; 11 Kenya Hospices and Palliative Care association (KEHPCA), Nairobi, Kenya; 12 City Cancer Challenge, Geneva, Switzerland; 13 Health Services Research & Policy, London School of Hygiene & Tropical Medicine, London, UK; a https://orcid.org/0000-0003-1417-7117; b https://orcid.org/0000-0002-4493-1216; c https://orcid.org/0000-0002-1294-9657; d https://orcid.org/0000-0001-5032-4714; e https://orcid.org/0000-0002-1337-8462; f https://orcid.org/0009-0003-4648-221X; g https://orcid.org/0000-0002-1022-576X; h https://orcid.org/0000-0003-3643-0304; i https://orcid.org/0000-0002-2351-4755; j https://orcid.org/0000-0001-9800-8067; k https://orcid.org/0000-0002-9755-3968; l https://orcid.org/0000-0003-3157-7067; m https://orcid.org/0000-0002-6435-1825

**Keywords:** palliative care, holistic care, global health, research productivity, citation impact

## Abstract

**Background:**

Palliative care research (PCR) plays a critical role in improving the quality of life for patients with serious illness, yet its global distribution and focus areas remain uneven. Understanding the trends and impact of PCR over the past decade can inform future research priorities and policy development.

**Methods:**

We conducted a bibliometric analysis of publications indexed in the Web of Science related to PCR between 2013 and 2022. Articles were identified using a comprehensive filter based on title keywords and specialist journals, and were further classified by research domain, disease area and study type.

**Results:**

The volume of PCR publications has grown over the past decade, increasing from 0.29% of all biomedical research outputs in 2013–14 to 0.62% in 2021–22. Countries with the highest levels of PCR output—primarily European and Anglophone nations—also ranked highly on the Economist Intelligence Unit’s Quality of Death Index. Using eight different bibliometric indicators, we assessed the impact of countries’ PCR outputs; while rankings varied by metric, European countries such as the Netherlands, Belgium, the United Kingdom and Ireland consistently performed strongly. Cancer emerged as the dominant disease focus, although many studies also addressed co-morbid conditions including COVID-19 in recent years. A significant proportion of PCR also examined the impact of illness on patients’ families and caregivers.

**Conclusion:**

The findings highlight cancer as a major area of focus and need within PCR. However, research outputs remain disproportionately concentrated in high-income countries, revealing a persistent gap in low- and middle-income settings.

**Recommendations:**

To address the growing global burden of cancer and serious illness, palliative care should be integrated as a core component of national cancer control plans. This integration must be supported by a targeted research agenda that emphasises implementation and scaling of palliative care models, particularly in low- and middle-income countries. Policymakers and research funders should prioritise holistic, patient-centred approaches and ensure that impact measurement reflects meaningful outcomes for patients and families.

## Introduction

Palliative care is intended to relieve the physical pain and mental distress associated with a severe illness without curative intent, and so to improve the patients’ quality of life. World Health Organisation has defined palliative care as ‘… an integrated approach that aims to improve the quality of life for patients—both adults and children—and their families facing the challenges associated with life-threatening illness. It focuses on the prevention and relief of suffering through early identification, thorough assessment, and effective management of pain and other physical, psychosocial or spiritual problems’ [1]. It is usually, but not always, provided for patients who are terminally ill, many with cancer [2]. Timely palliative care can be cost saving, improve quality of life and survival [3–7]. Research can identify more effective ways to treat patients and enhance the quality of life for those nearing the end of life [8]. It also encompasses areas such as patients’ preferences regarding advance care planning, their relationships with family members and the grieving process. In some countries, this research may include aspects related to assisted dying, where legally applicable. Palliative care is therefore a multidisciplinary field that spans both medical and sociological dimensions. Given the highly individual nature of patient needs, it is often described as holistic care and can be challenging to define in strictly clinical terms [9].

To date, bibliometric analysis has been applied to only a small proportion of palliative care studies—approximately 0.1%—highlighting an opportunity to further explore research trends and gaps within the field. The most recent one, by researchers in Taiwan [10], covered the period from 2001 to 2016 and also analysed the citations to the papers. However, their search strategy was very limited in scope and identified a small number of papers. Some 50%–60% of papers germane to palliative care were not picked up by their limited search strategy in comparison to the more advanced one used in this analysis. An earlier analysis spanning the 21 years from 1993 to 2013, also from Taiwanese researchers [11], identified many more papers in the Web of Science in and found a very rapid growth rate (13%) in the field across that time period. There have also been studies of research activity (bibliometric studies) in palliative care in the continents of Africa [12], Asia [13] and Latin America and the Caribbean [14] as well as commentary on the disparities in low- and middle-income countries (LMICs) contributions to palliative care research (PCR) [15]. The Cheong study [13] compared research outputs with the Economist Intelligence Unit’s (EIU) 2015 Quality of Death Index (QODI), [16] encompassing 40 countries only and found a good correlation (R = 0.85), but many Asian countries carried out little or no relevant research. The Rhee study [12] found very few PCR papers from Africa (*n* = 47) published between 2005 and 2016, which may have seriously under-estimated work in that continent, such as the rapid expansion of palliative care provision in South Africa and Uganda [17]. Moreover, this search only focused on palliative care papers describing the development of services rather than wider literature. These reports and the more recent Global Atlas of Palliative Care [18] paint a comprehensive picture of the current provision of palliative care in different countries and regions of the world but also the need for research to shape that care. The situation is certainly improving, but huge gaps remain especially in less-developed nations.

The EIU report takes a more detailed approach, dividing the QODI into five categories:

- The palliative and healthcare environment
- Human resources and the need for training
- The affordability of palliative care
- The quality of care and
- Community engagement

Several of these map onto the research portfolio, the subject of this paper, which has been divided into some 19 research domains (Table 1). These research domains have been derived from standard bibliometric ‘research’ headings that are part of standard MESH descriptors of published papers. These are broad topic headings that have been established and validated by bibliometric databases that describe the type of research domain to which the paper is primarily concerned. Seven of these are diseases or conditions from which patients receiving (or needing) palliative care may suffer, but the others relate to the training of healthcare workers or are sociological aspects such as the effects on the patients’ relatives and the costs of care.

In addition, research may also be an indicator of the progress of palliative care in a country [19, 20]. Research advances knowledge and evidence-based practices, informs clinical guidelines and policies that ensure patients receive consistent, high-quality care across various settings [21, 22]. PCR identifies best practices, effective interventions, captures the complexity of patients, symptoms and concerns, informing holistic and innovative care models that can improve patient outcomes [22]. PCR informs what is feasible, accessible and effective palliative care and how best to develop and implement the most relevant and sustainable services [23]. Research is key in generating evidence across settings on early and timely integration of palliative care in the treatment process, on how to customise referral criteria, leading to better symptom control, reduced hospitalisations and enhanced patient and family satisfaction on a global scale [24]. The Lancet Commission on Palliative Care and Pain Relief Report highlights the need for health systems research, specifically implementation research on lessons learned from country experiences on both successful and failed programs particularly those impacting high-risk populations such as victims of humanitarian emergencies, migrant communities and children [25].

Despite the growing importance of palliative care as a global health priority, there remains a limited understanding of the scope, distribution and thematic focus of PCR worldwide. Understanding key areas and trends in PCR is essential for guiding research and funding priorities [26], particularly to ensure that under-researched areas—such as paediatric palliative care—receive adequate attention and support [27]. However, few studies have systematically mapped this evolving research landscape.

This study addresses that gap by asking: ‘What are the global trends, thematic priorities, and geographical patterns in PCR over the last decade, and how can these insights inform future research, policy, and education?’ To answer this question, we conducted a bibliometric analysis of PCR publications indexed in the Web of Science from 2013 to 2022. Articles and reviews were identified using a comprehensive filter based on title keywords and specialist journals and further categorised by disease area and study type.

Bibliometric analysis offers a robust, data-driven approach to evaluate the volume, distribution and impact of research outputs across time and geography [28]. By identifying underrepresented topics and regions and mapping patterns of collaboration and authorship, it provides objective evidence that can inform strategic planning and advocacy efforts in palliative care.

Findings from this work have multiple applications. Insights into prevalent themes—such as pain management—can support the development of evidence-based health policies, help clarify implementation strategies and contribute to the standardisation and scaling of palliative care services to improve access and quality [29]. Furthermore, the analysis can inform the design of education and training programs, ensuring healthcare professionals are equipped with up-to-date knowledge and best practices [30–32].

Importantly, identifying regional contributions can foster international collaboration and knowledge exchange while also exposing disparities in research output that may guide global health policies toward more equitable access to palliative care [33]. These insights can help shape future research agendas [34], promoting interdisciplinary and patient-centred approaches that prioritise the quality of life.

Several different groups may benefit from the outcomes of this analysis. Policymakers can use the findings to allocate resources more effectively and to support service development in under-resourced areas. The results also provide an evidence base for the creation of prioritised research agendas at national, regional and global levels. Moreover, the study offers opportunities to promote new partnerships between high-performing and developing regions, fostering capacity-building through training and resource sharing. Ultimately, by identifying emerging research areas, gaps and global patterns, this bibliometric analysis contributes to the strategic development of more inclusive, accessible and high-quality palliative care systems worldwide.

## Methodology

In carrying out a bibliometric study of PCR, we relied mainly on the titles of the papers in the Web of Science (WoS; © Clarivate Analytics), but also on a few specialist journals, the large majority of whose papers were deemed relevant to the subject.

We co-developed a filter (available upon request from the corresponding authors), containing 51 title words and phrases and 13 specialist palliative care journals, that was applied to the WoS for the 10 years, 2013–22. This used pre-existing palliative care search terms supplemented by expert input and iterative search processes to refine the search strategy. The bibliographic data from articles and reviews thus identified were downloaded to Excel files and processed by a special macro (computer program) to create a single spreadsheet. Several Boolean conditions were needed to limit the application of some words or phrases in the filter. For example, *end of life* appears in the title of numerous papers concerned with the scrappage of vehicles and solar panels, as well as in many medical papers, which would be relevant to palliative care. We calibrated the filter [35] with reference to papers with and without an appropriate address word such as *hospice* or *palliative*, and found the precision, p, to be 0.90 and the recall, r, to be 0.85. This means that the true number of palliative care papers would have been p/r = 1.064 times the apparent number. Subsequently, 17 papers were found to be about the euthanasia of farm animals and were removed. The total of 33,593 papers also included 807 papers published in print in 2023 that appeared in online versions of journals in the previous year. These were included in the analysis of research domains, but not in the yearly tally of papers from different countries.

Further macros enabled us to mark each paper with the fractional counts of each country represented among the addresses. For example, a paper with two addresses in France and one in the USA would be categorised as FR = 0.67, US = 0.33, but as FR = US = 1 on an integer count basis. Country PCR outputs for 2013–17 were plotted against the country Gross Domestic Product in 2015, and also against the EIU QODI for that year [16] in order to see if good practice in the provision of palliative care was correlated with the amount of research. We also compared PCR outputs with the countries’ biomedical research outputs [36] in the two five-year periods, 2013–17 and 2018–22. [Incidentally, these latter decreased in the last 2 years in most countries because of COVID-19, so that the 5-year totals also decreased].

We also marked each paper to show if it could be regarded as being within each one of some 19 different research domains. These were given short codes (four or five letters) for ease of reference, see Table 1. Their presence in a domain was determined from words in their titles. The fractional count of each of the leading 32 countries in each domain was marked, and their relative commitment to each domain (relative to their presence in PCR) was then calculated as a decimal, greater or smaller than unity.

We wished to compare the countries based on the impact or utility of their PCR outputs. For this purpose, we used five different indicators commonly used in bibliometric analysis:

1. The citation impact factor of the journals (JIF) in which their papers were published. These factors have been determined for most of the journals (but not all) and published by Clarivate Analytics. Although they are an imperfect measure of influence, they do correlate quite well with other measures such as a mean citation score in a fixed time window (we used 5 years). We called this Actual Citation Impact, ACI.
2. The geometric mean is a better measure than the arithmetic mean because the distribution of citation numbers is unusual statistically, with a few papers receiving very high numbers of cites (several hundred) but most, fewer than ten. This can be calculated if unity is added to each citation score, the natural logarithm is taken, the mean for a group is then calculated and the result exponentiated and unity subtracted [37]. The resulting measure is typically half the arithmetic mean.
3. The fractional count of the number of papers whose citation score is greater than the number needed to put them in the top-cited 5% of all PCR papers in 2013–18, which was 38. In fact, 814 papers or 5.023% achieved this score as citations are integers. The resulting percentage, divided by 0.05023, was called World Scale (WS) by analogy with world oil tanker charter rates [38].
4. The percentage of reviews, as classified by the WoS; a mark of esteem in which the country’s authors are held by journal editors as these documents are usually commissioned by them from leading experts in the subject [39].
5. A relatively new measure, the mean number of downloads of PCR papers since 2013. Since this obviously decreases for more recent papers (there has been less time for them to be downloaded), the mean score for each country and year was normalised with respect to the mean for the world total. This decreased from 16.0 for 2013 papers to 5.0 for ones published in 2022 [40, 41].

Because international papers tend to be more frequently cited than domestic ones for a given country, and their mean citation score is likely to be affected by the prowess of their foreign partners, we also calculated this for the leading countries’ domestic papers as an alternative indicator. We made this distinction for the indicators based on JIF, on ACI and on downloads, so making eight in total. This approach provides a very comprehensive comparison of the quality of the PCR of the leading countries in addition to simpler metrics of impact, e.g., output relative to a country’s wealth.

## Results

**Figure 1.**
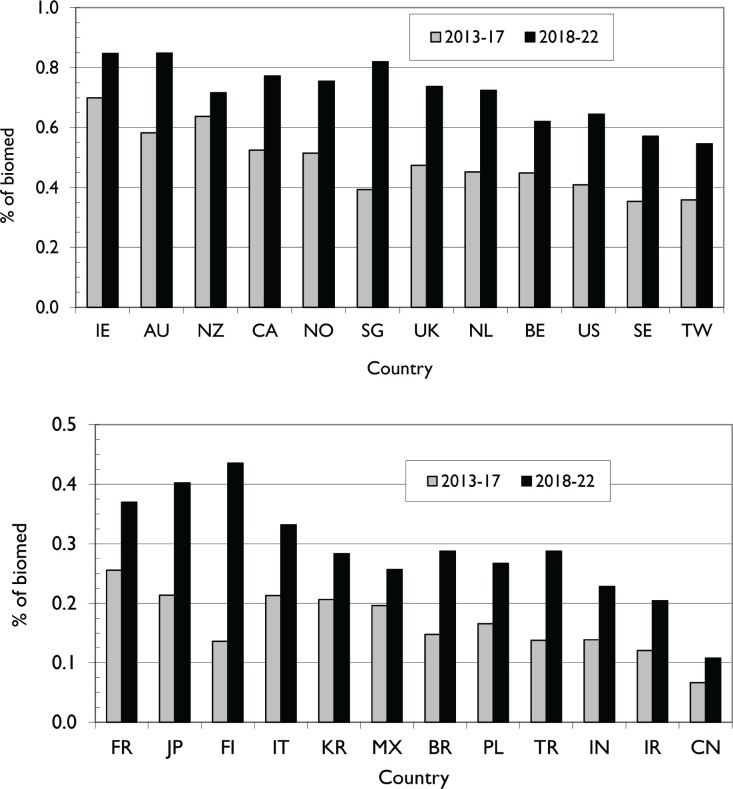
(a): The percentage of biomedical research attributable to PCR from 12 leading countries in two five-year periods: highest percentages. IE = Ireland, AU = Australia, NZ = New Zealand, CA = Canada, NO = Norway, SG = Singapore, NL = Netherlands, BE = Belgium, SE = Sweden, TW = Taiwan. (b): The percentage of biomedical research attributable to PCR from 12 leading countries in two five-year periods: lowest percentages. FR = France, JP = Japan, FI = Finland, IT = Italy, KR = South Korea, MX = Mexico, BR = Brazil, PL = Poland, TR = Türkiye, IN = India, IR = Iran, CN = China.

In the 10-year study period, there were 32,786 published PCR papers, which represented only 0.4% of all biomedical research during those years. However, this percentage rose from 0.3% in the first 6 years to attain 0.6% in 2022, when overall biomedical output was declining, probably because of the effects of COVID-19. Countries varied greatly in their relative commitment to PCR, see Figure 1a and b. Ireland (IE), Australia (AU) and New Zealand (NZ) showed the highest value, and the first two, with Singapore (SG), all published more than 0.8% of their biomedical research outputs on PCR in 2018–22. At the other extreme, in addition to countries with no publications, four Asian countries China (CN, 0.09%), Iran (IR, 0.16%), India (IN, 0.18%) and Türkiye (TR, 0.21%) all published much less PCR than the world mean over the 10 years.

**Figure 2.**
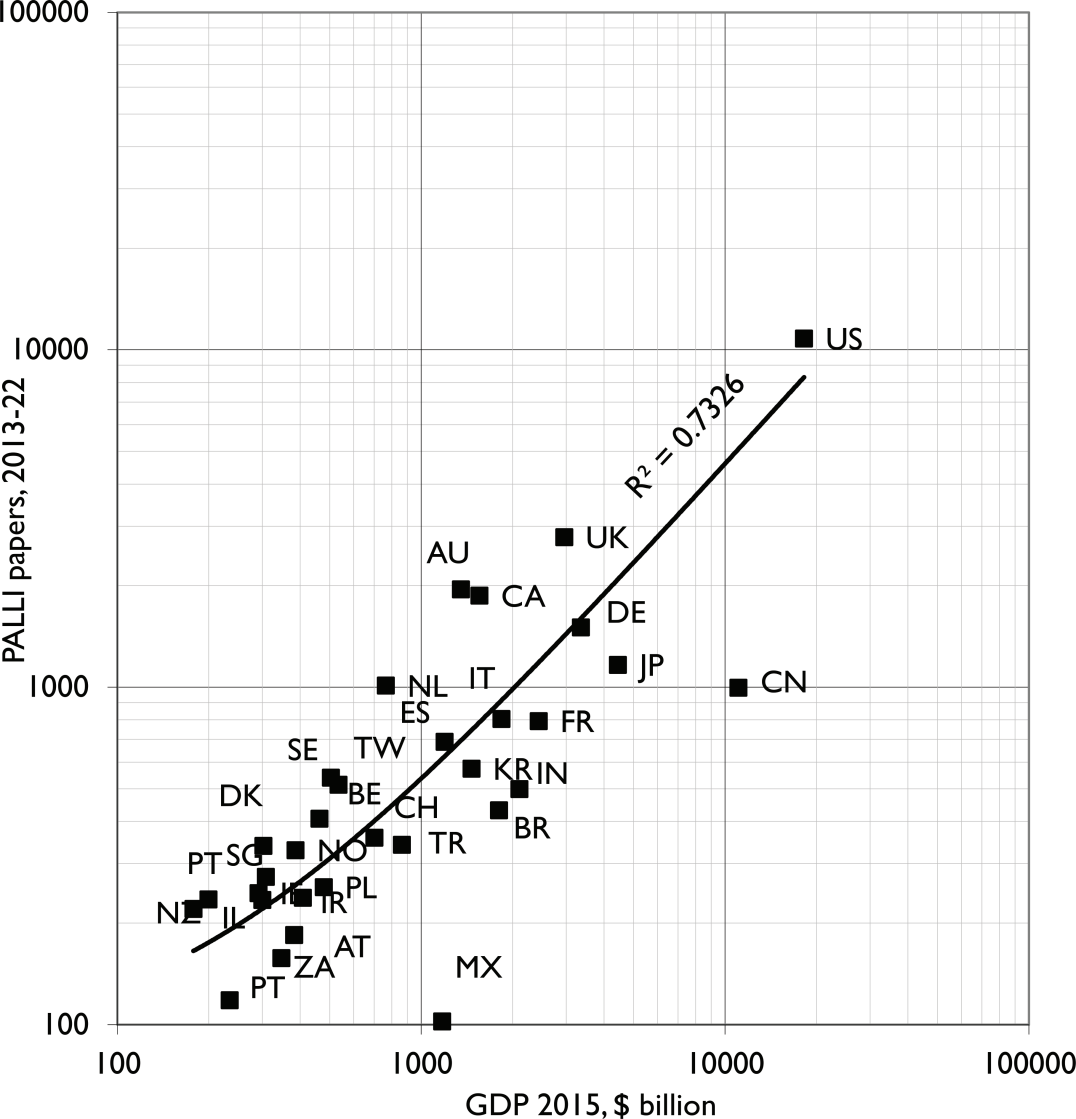
Plot of PCR outputs in 2013–17 for leading countries against their Gross Domestic Product in 2015 (World Bank data). Digraph codes as in Figure 1a and b; Also AT = Austria, CH = Switzerland, DE = Germany, DK = Denmark, ES = Spain, IL = Israel, PT = Portugal, ZA= South Africa.

Some countries made big increases in their PCR publication share, notably Singapore, the Netherlands (NL) and the United States among those with the highest relative commitments, and Finland (FI), Brazil (BR) and Türkiye among the laggards. As expected, the amount of PCR in 2013–17 from the different countries correlated much better with GDP in 2015 (r^2^ = 0.73) than with population (r^2^ = 0.19). The graph with GDP (Figure 2) shows that China (CN) and Mexico (MX) are by far the lowest performers.

**Figure 3.**
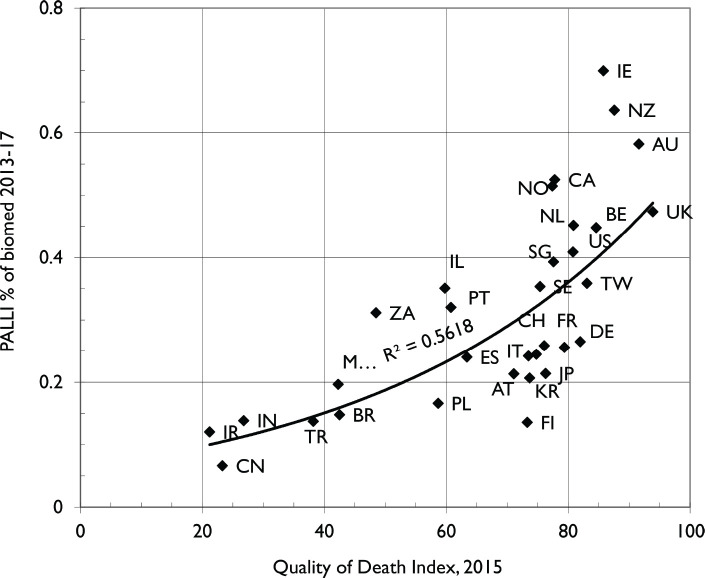
Comparison of the amount of PCR relative to the total biomedical research in 2013–17 of leading countries with the EIU QODI for 2015.

We then investigated whether the amount of PCR correlated with the ‘QODI’. This is shown in Figure 3. The correlation is strongly positive. The two main outliers in Figure 2, China and Mexico, have spots that are now close to the best-fit curve. The new outliers are Finland (FI), which did relatively little PCR compared with its QODI ranking but substantially increased it in 2018–22, see Figure 1b, and Ireland (IE) and New Zealand (NZ), which perhaps devoted more effort to PCR than was needed. However, our selection of countries excludes, for example, Uganda, a low-income country that ranked well in the QODI and above India a low-middle and just below Brazil an upper–middle income country.

The division of PCR into the disease domains was dominated by cancer research (29.7%), followed by mental disorders (8.2%), cardiovascular research (3.5%) and renal medicine (3.1%). However, in the last 3 years (2020–22), research on these last two disease areas was overtaken by research on patients with COVID-19. AIDS as a disease area attracted very little PCR except in South Africa (ZA), where the relative commitment was over 16 times the world norm, and the USA. Diabetes only led to 0.3% of PCR.

Of the other research domains listed in Table 1, the most popular was the effects on family members and friends, with 13.5% of the PCR total. Other domains were research on children (7.3%), which was very similar to the proportion of people needing palliative care each year (about 57 million) who were children (7% at least) [17]. Dealing with pain and concern with quality of life each attracted 4.6% of the total, followed by ethical considerations (3.4%) and the education and training of healthcare workers (3.0%). Since the QODI allocated one fifth of its score to this category, this seems to be unduly low. There was also very little PCR on the costs of treatment. Since the QODI report noted that more attention to the provision of palliative care could lead to greater savings elsewhere in a country’s health service costs, this also seems to be a notable omission.

The last three research domains were the three main treatment methods: use of medicines (5.2%), radiotherapy (2.7%) and surgery (2.2%). Over 500 papers simply referred to ‘chemotherapy’, but most papers on medicines mentioned one or more individually named products. The ones most commonly listed were methadone (*n* = 48 papers, 0.14%), paclitaxel (*n* = 23) and cisplatin (*n* = 20).

Tables 2 and 3 show the relative commitment of the 32 leading countries to each of the research domains in PCR. Their outputs are fractionated, and the values are presented relative to unity, which is the world value. Values over 4 are in cells tinted dark green, over 2 in cells tinted mid-green, over 1.414 (=√2) tinted pale green, below 0.707 (=1/√2) tinted pale yellow, below 0.5 tinted light brown and equal to zero tinted pink. For the larger countries scientifically, the values depart less from unity but there are some interesting exceptions. Thus, the USA has a big PCR effort on AIDS, where the disease first drew major attention, but much less on diabetes as a cause of the need for palliative care. The latter occasioned more PCR in Taiwan (TW), and in Singapore (SG), Australia (AU) and China (CN). PCR for cancer was actively pursued in East Asian countries such as Japan (JP), South Korea (KR), China (CN) and Taiwan (TW).

Of the other research domains, PCR on the effects on family members is most notable in the three Scandinavian countries but not Finland (FI), although it increased its output markedly in the later quinquennium. The use of medicines in palliative care was researched particularly in the east Asian countries (Japan (JP), South Korea (KR) and China (CN)), but not Taiwan (TW) and definitely not Singapore (SG). These three countries also researched the use of palliative radiotherapy and surgery. Belgium (BE) had much the highest relative commitment to ethical issues, followed by the Netherlands (NL) and Spain (ES). Almost all the primarily Belgian papers in this domain were about euthanasia, which was legalised in 2002, even for children of all ages, and de-criminalised assisted dying, but had some definitional problems [42]. Belgium, with the Netherlands, also did relatively the most research on advanced directives (ADVA), which can prescribe the limits on treatment of a patient who may be unable to speak. In view of the lack of research attention to the training of healthcare workers, there are exceptions in Ireland (IE) and Finland (FI). These papers cover many levels of personnel from doctors and nurses to pharmacists and nursing home health-care assistants.

We turn next to the measures of the impact of PCR. Table 4 gives the actual values of the different parameters, except for the downloads, which are the ratio of the numbers for individual papers to the mean for the world in that year and so are relative to unity. The ranking of the leading 32 countries on each of the eight parameters, and overall, is shown in Table 5. It is striking that there is considerable divergence between these rankings. For example, the Netherlands (NL) and Belgium (BE), which clearly rank at the top, do not score highly for the percentage of reviews (% rev.). On the other hand, Ireland (IE), which ranks first for the percentage of reviews and for its WS value, only published its papers in low-impact factor journals. China’s (CN) papers were the most downloaded by a large margin but were in less-cited journals and were not well cited. However, this high ranking meant that the overall rank of China was much higher than that of most other Asian countries. The ranking of countries’ domestic papers often differed markedly from that of all their papers. Thus, the UK ranked much higher on JIF and ACI for its domestic papers, but Switzerland (CH) showed the reverse. However, the ranking of downloads (for which r^2^ = 0.88) altered relatively much less than for JIF (r^2^ = 0.17) and for ACI (r^2^ = 0.46).

A similar analysis was performed on the PCR papers in the 19 different domains and the results are shown in Table 6. The two formats (actual values and rankings) have been shown in the left and right tables, respectively, with similar cell tinting as in Table 4.

Because none of the papers on COVID-19 had citation scores, a mean rank of 10 was added to their rank on the three other parameters so that their impact could be compared with the papers in the other domains. It appeared that papers on ADVA and on cancer (ONCOL) had the greatest influence. Papers on the training and education of healthcare personnel (EDUC) ranked poorly, which may be discouraging for work on this subject which is important for the overall ranking of countries as providers of a good death.

## Discussion

Annually, around 60 million people experience serious health-related suffering that affects physical, social, spiritual and emotional well-being and requires professional intervention [26]. This trend is expected to continue for several decades, mainly affecting LMICs due to a disproportionate rise in rates of cancer, cerebrovascular disease, lung disease and dementia [43]. Despite the increasing need, palliative care, and the associated research, continue to be relatively neglected areas of medicine, especially in less-developed countries [44]. Whilst the PCR share of all biomedical research more than doubled between 2013 and 17, the overall levels of research remain well below what is needed to address the global burden.

While countries such as Ireland, Australia, and Singapore have demonstrated increased activity in PCR, others—including China, Iran and India—show comparatively lower levels of research output within the period analysed. The relationship between healthcare spending and the economic performance of countries is stable over time with occasional sudden changes and breaks in their trends [45]. Clark *et al* [46] observed moderate to strong associations between indicators of national development, including the percentage of a country’s GDP spent on public health expenditure, corruption, and weak governance, with the level of palliative care development. Our findings align with these observations, where countries with higher GDPs generally produced more PCR. This indicates that economic resources significantly influence research capacity in PCR, with China and Mexico notably underperforming relative to their GDPs. Other factors such as the relative level of development of palliative care may also play a role in PCR; for example, Mexico is at an early stage of ‘isolated provision’, characterised by limited support for activism, donor-dependent funding, scarce morphine availability and small-scale service provision [47].

Our findings correlate surprisingly well with the QODI. There is, however, remarkable heterogeneity around the quality of PCR on a country-by-country basis. The positive correlation between PCR and the QODI further validates the importance of PCR in enhancing the quality of end-of-life care. Outliers such as Finland, which showed less PCR relative to its QODI ranking but increased its output substantially in recent years, indicate potential areas for strategic research investment. Conversely, countries like Ireland and New Zealand, with higher PCR than their QODI might suggest a strong commitment that perhaps exceeds immediate national needs. Both Ireland and New Zealand have palliative care services at an advanced stage of integration to mainstream healthcare services, with widespread palliative care activism, comprehensive service provision, broad awareness and an updated strategy [47].

Cancer research leads PCR, followed by mental disorders, cardiovascular diseases and renal medicine. This is not unexpected as cancer poses a significant burden in both high-income countries and emerging economies [47–50]. Life-limiting illnesses vary significantly across different regions due to varying prevalence of specific diseases and health conditions. Approaches to palliative care—and by extension, PCR—vary significantly across countries, often reflecting national disease burdens and healthcare priorities. In high-income countries, non-communicable diseases such as cancer, cardiovascular diseases and dementia are predominant causes of life-limiting conditions [48]. In LMICs, communicable diseases like HIV/AIDS and tuberculosis are more prevalent, although non-communicable diseases are also on the rise [51] and projections estimate that by the year 2040 as much as 70%–75% of cancer mortality will be in LMICs [22]. This divergence in clinical focus may influence the nature and scope of research activity across regions. For instance, in settings where palliative care is still primarily associated with cancer, research into other domains may be under-prioritised. Our findings also highlight the variability in PCR focus across countries and therefore relative readiness to respond to this growing need for cancer-related palliative care services. Notably, South Africa and the USA had a higher relative commitment to AIDS-related palliative care, reflecting regional disease burdens and healthcare priorities. In contrast, East Asian countries like Japan, South Korea and China focus more on palliative care related to cancer and the use of medicines. Belgium and the Netherlands show a strong emphasis on ethical issues, particularly euthanasia, aligning with their progressive healthcare policies. Scandinavian countries, excluding Finland, lead in research on the effects of palliative care on family members, while Ireland and Finland show notable attention to the training of healthcare workers. Our analysis also reveals that countries like the Netherlands and Belgium, despite not publishing extensively in journals with high JIF values, achieve high citation metrics, indicating influential research. Ireland’s high ranking in review publications suggests a leadership role in synthesising and guiding PCR knowledge. Meanwhile, China’s high download rates reflect substantial global interest in its PCR outputs, although its citation metrics indicate room for quality improvement. These findings highlight the varied national priorities, capacity and potential areas for international leadership, collaboration and knowledge sharing. These include the evidence of performance, outcomes and cost-effectiveness of innovative models of care to shape and drive scale-up of these critical services especially in LMICs with equity of access, integrated models of care and quality of services in focus.

## Conclusion

This comprehensive analysis presents the evolving landscape of PCR and its critical role in global healthcare. The disparities in national research output and focus areas reveal opportunities for targeted investments and international collaboration. As the demand for high-quality palliative care continues to grow, especially in the wake of global health challenges such as COVID-19 and cancer, fostering a balanced and comprehensive approach to PCR is essential. There remain huge gaps in research into critical domains that will inform national policy such as service delivery models in LMICs and quality improvement of these services. Countries must not only increase their research output but also ensure alignment with global best practices and healthcare needs to improve the quality of life for patients with advanced and life-limiting illness. Addressing the identified gaps and disparities is crucial for improving the quality of palliative care worldwide, ensuring that research efforts are more equitably distributed and aligned with the diverse needs of different populations.

## Conflicts of interest

None.

## Consent for publication

Not applicable.

## Ethics approval and consent to participate

Not applicable.

## Author contributions

Study conception and design: MBH, GL, RS. MBH is the lead of the study and JT the coordinator. Analysis and interpretation of results: ALL carried out the first analysis, ALL contributed to further interpretation. Draft manuscript preparation: RS and MBH drafted the first draft of the paper. ALL provided critical feedback. ALL reviewed the results and approved the final version of the manuscript*.*

## Availability of data and materials

Requests for relevant codes and other relevant materials for search can be made through the corresponding author. This request will be reviewed by the principal investigator (RS).

## Figures and Tables

**Table 1.** Nineteen research domains within PCR linked to oncology and codes.

Code	Description
ADVA	Advance care directives
AIDS	HIV & AIDS research
CARDI	Cardiology research, including stroke
COST	Access to care, medicare, billing, costs
COVID	COVID-19 research
DIABE	Diabetes research
MEDI	Palliative treatment with medicines and chemotherapy
EDUC	Education and training of healthcare workers
ETHI	Ethical issues, including assisted dying and euthanasia
FAMI	Family reactions to the patient and prognosis
INDI	Indicators
MENTA	Mental health research
ONCOL	Cancer research
PAED	The care of infants and children
PAIN	Treatment of pain and use of opioids
QUAL	Quality of life and survivorship
RADI	Radiotherapy for palliation
RENAL	Renal medicine research
SURG	Surgery for palliation

**Table 2.** Relative commitment of 32 leading countries to disease domains within PCR. Domain codes are in Table 1; country codes are below Figures 2a,b and 3.

	ONCOL	MENTA	CARDI	RENAL	COVID	AIDS	DIABE	PALLI
US	0.85	1.03	1.37	0.91	1.00	1.69	0.43	**11,103**
UK	0.62	1.14	0.94	0.78	1.51	0.68	0.76	**2,857**
AU	0.74	1.09	0.33	0.83	0.72	0.43	2.50	**1,998**
CA	0.92	0.93	0.91	0.61	0.41	0.55	1.75	**1,909**
DE	1.01	0.89	0.82	0.47	1.09	0.18	0.21	**1,528**
JP	2.06	0.40	1.29	0.86	0.27	0.23	0.94	**1,188**
NL	0.92	1.65	0.68	0.80	1.06	0.15	0.48	**1,034**
CN	1.75	0.53	1.06	2.20	0.85	0.55	2.32	**1,033**
IT	1.50	0.81	1.41	0.99	1.46	0.32	0.91	**820**
FR	1.00	0.55	0.28	0.81	0.35	0.02	0.63	**795**
ES	0.72	1.27	0.59	0.45	1.42	1.26	0.53	**699**
KR	1.83	0.47	0.99	2.04	0.09	0.49	0.64	**587**
SE	0.86	0.73	1.61	0.90	0.78	0.09	2.33	**549**
TW	1.53	0.69	1.15	4.62	0.63	0.52	5.51	**522**
IN	1.42	0.57	0.55	1.22	2.79	0.46	0.74	**508**
BR	1.15	0.85	0.44	1.01	2.07	0.60	0.00	**447**
BE	0.64	1.12	0.67	0.17	0.34	0.98	0.00	**416**
CH	0.78	1.06	0.93	0.31	0.48	0.00	0.60	**363**
TR	1.20	0.96	0.82	1.05	0.89	0.00	1.04	**360**
DK	1.21	1.20	1.02	0.90	0.60	0.00	2.35	**351**
NO	1.25	0.93	0.23	0.13	0.43	0.16	1.19	**337**
SG	1.09	0.56	1.17	1.38	0.90	0.48	3.31	**282**
PL	0.99	0.65	1.02	1.62	0.23	0.00	1.52	**256**
IE	0.66	1.53	0.54	0.60	1.19	0.00	0.00	**255**
IR	1.23	1.12	0.80	1.22	2.57	0.00	0.00	**246**
IL	0.88	2.98	0.41	0.29	0.34	0.00	1.14	**245**
PT	0.53	1.25	0.75	1.19	1.48	1.12	2.52	**239**
NZ	0.33	0.67	0.37	0.54	0.97	0.63	1.65	**227**
n.a.	0.27	3.60	0.78	0.45	0.95	0.00	1.73	**216**
AT	1.12	0.32	0.70	0.59	0.53	0.00	0.00	**188**
ZA	0.72	1.32	0.24	1.64	0.70	16.6	0.00	**159**
FI	0.84	1.42	0.49	0.05	0.00	0.00	0.63	**123**
MX	1.20	1.88	0.89	1.73	1.80	0.00	6.32	**103**
	**9,749**	**2,766**	**1,191**	**1,046**	**652**	**125**	**90**	**3,3592**

n.a. = no address given

**Table 3.** Relative commitment of 32 leading countries to all other domains within PCR. Domain codes are in Table 1; country codes are below Figures 2a, b and 3 and listed in the same order as Table 2.

	FAMI	PAED	MEDI	QUAL	PAIN	ADVA	ETHI	EDUC	RADI	COST	SURG	INDI
US	0.93	1.28	0.76	0.96	1.25	1.33	0.68	1.27	0.80	1.45	1.23	0.65
UK	1.28	1.10	0.62	0.72	0.68	0.98	0.75	1.14	0.53	0.94	0.43	1.00
AU	1.19	0.85	0.80	0.71	0.64	1.90	1.04	0.82	0.59	1.00	0.27	0.29
CA	0.96	0.89	0.91	1.10	0.99	1.04	0.99	0.87	2.07	1.40	0.55	1.36
DE	0.81	0.89	0.98	0.91	0.99	0.53	1.27	0.72	1.07	0.66	1.09	1.14
JP	0.93	0.36	2.49	1.12	1.56	1.02	0.08	1.00	2.77	0.65	2.61	1.83
NL	1.27	0.78	1.34	1.27	0.43	1.56	2.23	0.79	0.90	0.67	0.54	1.96
CN	0.96	0.52	2.18	1.21	1.09	0.83	0.12	0.88	1.86	0.37	2.05	0.72
IT	0.62	0.68	1.60	1.18	1.45	0.38	1.15	0.52	1.99	0.63	1.25	0.83
FR	0.77	1.35	1.28	0.64	0.92	0.05	1.27	0.50	0.51	0.63	0.80	0.64
ES	0.80	0.73	1.02	0.85	0.76	0.23	2.25	1.06	0.44	0.41	0.30	2.95
KR	0.97	0.39	2.24	1.26	0.63	0.62	0.35	0.80	0.77	0.79	1.92	2.26
SE	1.76	0.96	0.88	1.27	0.51	0.27	0.47	1.10	0.29	0.49	0.69	1.57
TW	1.17	0.26	0.84	1.12	0.29	1.65	0.22	0.67	0.33	1.03	1.36	1.46
IN	0.44	0.84	1.94	1.39	1.46	0.09	0.55	0.52	3.01	0.54	1.37	0.00
BR	0.85	0.75	0.74	1.51	0.88	0.08	1.33	1.09	0.47	0.47	0.73	0.81
BE	0.86	0.77	0.62	1.55	0.35	2.20	4.70	0.56	0.46	0.78	0.31	7.40
CH	0.70	1.28	1.09	0.53	0.71	0.91	1.35	0.55	0.91	0.83	0.24	0.66
TR	1.28	0.94	0.99	1.43	1.14	0.02	1.09	0.86	0.82	0.27	1.42	0.00
DK	1.91	0.62	0.93	0.97	0.89	0.57	0.36	0.59	0.47	0.59	0.26	1.62
NO	1.81	0.80	1.11	1.09	1.50	1.08	0.73	0.40	5.42	0.21	0.40	1.02
SG	1.31	0.84	0.39	1.38	0.41	2.00	0.35	0.95	0.93	1.22	1.51	0.26
PL	0.33	0.63	2.37	1.49	1.94	0.08	1.15	0.56	1.58	0.86	1.35	0.85
IE	1.37	1.11	0.51	0.93	0.31	1.15	0.62	2.13	0.62	1.22	0.33	0.85
IR	0.86	1.81	1.33	1.86	2.22	0.00	2.06	1.20	0.45	0.33	0.37	0.44
IL	2.04	0.84	0.67	0.48	0.37	0.98	1.30	0.31	0.42	0.18	0.59	1.19
PT	1.14	0.71	0.77	0.86	0.77	0.22	0.98	0.73	0.62	0.19	0.22	0.00
NZ	1.34	0.33	0.73	0.29	0.28	0.69	2.69	1.23	0.28	1.77	0.36	0.96
n.a.	0.74	0.77	0.09	0.20	0.00	0.40	2.73	0.62	0.34	0.20	0.00	0.00
AT	0.79	0.83	1.38	0.25	0.83	0.27	2.79	1.33	0.50	0.20	1.00	0.29
ZA	1.35	2.08	0.41	0.59	0.44	0.10	1.80	1.34	0.22	0.87	0.58	3.94
FI	0.84	0.75	0.38	0.92	0.62	0.82	3.30	2.87	0.36	1.21	0.75	0.00
MX	0.49	1.22	0.41	1.39	0.47	0.05	2.68	0.04	0.00	1.96	1.19	1.05
	**4,549**	**2,437**	**1,738**	**1,555**	**1,551**	**1543**	**1,139**	**1,011**	**912**	**765**	**728**	**154**

**Table 4.** Impact parameters for PCR papers, 2013–22, for the 32 leading countries.

Impact	JIF all	JIF dom.	ACI all	ACI dom.	WS 5%	% rev.	DL all	DL dom.
AT	3.92	2.53	4.89	3.16	67	12.6	0.852	0.742
AU	3.45	2.69	8.71	7.05	99	16.7	1.238	1.072
BE	4.01	3.27	10.40	7.28	149	12.5	1.385	1.257
BR	2.72	1.91	4.54	3.36	37	19.4	0.891	0.807
CA	3.83	2.95	8.66	6.68	110	14.7	1.056	0.918
CH	4.69	2.84	9.26	4.92	95	15.5	1.179	0.889
CN	3.38	3.03	7.27	6.38	70	14.5	1.958	1.93
DE	3.38	2.5	6.16	3.85	82	11.1	1.003	0.863
DK	4.21	2.91	9.15	7.74	75	10.3	1.086	1.005
ES	3.48	2.58	6.81	3.90	78	12.0	1.234	1.143
FI	3.54	2.77	8.25	6.07	27	9.4	1.167	1.111
FR	4.06	2.82	3.34	1.71	60	8.6	0.715	0.521
IE	3.59	2.74	9.51	5.54	168	20.3	1.241	1.18
IL	3.89	2.47	6.76	3.67	66	6.8	1.359	1.166
IN	4.17	2.72	3.86	2.73	17	14.7	0.7	0.465
IR	2.64	2.4	3.54	3.28	0	10.7	0.98	0.883
IT	3.92	3	9.15	6.20	122	14.1	1.033	0.968
JP	3.21	2.77	5.39	4.64	41	6.5	0.755	0.676
KR	3.18	2.72	7.12	5.32	51	6.9	1.012	0.872
MX	3.56	2.02	3.19	1.39	17	12.1	0.93	0.547
NL	4.24	3.55	12.01	10.08	127	10.8	1.401	1.209
NO	3.71	2.73	9.91	7.85	124	11.1	0.986	0.807
NZ	2.79	2.51	7.16	6.01	77	14.0	1.082	1.013
PL	4.29	2.54	2.74	1.61	23	16.9	0.751	0.552
PT	2.84	2.06	6.16	4.41	32	16.7	1.147	1.033
SE	3.32	2.78	8.09	6.91	103	7.2	1.115	1.066
SG	3.49	2.99	6.80	5.47	54	15.0	1.133	0.99
TR	2.37	1.74	3.66	2.79	4	7.1	1.125	1.069
TW	3.2	2.8	6.32	5.57	18	4.7	1.189	1.006
UK	3.84	3.3	8.92	7.22	114	19.1	1.166	1.064
US	3.57	3.27	7.73	7.04	124	10.1	0.998	0.929
ZA	4.33	2.23	5.53	2.42	34	13.2	0.885	0.592

DL = downloads since 2013, divided by the world mean for each year. Dom = domestic papers without international partner. Values > 1.414 × world mean tinted pale green; if < 0.707 × world mean, tinted pale yellow; if < 0.5 × world mean, tinted light brown

**Table 5.** Ranking of 32 leading countries in PCR impacts according to the above table for each of eight parameters and overall (right-hand column).

Rank	JIF all	JIF dom.	ACI all	ACI dom.	WS 5%	% rev.	DL all	DL dom.	Total	Rank
NL	4	1	1	1	3	21	2	3	36	1
BE	8	3	2	4	2	16	3	2	40	2
UK	12	2	8	5	7	3	11	11	59	3
IE	15	16	4	15	1	1	5	4	61	4
CH	1	10	5	18	11	7	9	20	81	5
CN	23	5	14	10	16	11	1	1	81	6
AU	21	20	9	6	10	5	6	8	85	7
IT	9	6	6	11	6	12	19	17	86	8
DK	5	9	7	3	15	23	16	15	93	9
CA	13	8	10	9	8	9	18	19	94	10
NO	14	17	3	2	4	19	23	24	106	11
US	16	4	13	7	5	24	22	18	109	12
SE	24	13	12	8	9	26	15	10	117	13
SG	19	7	18	16	20	8	13	16	117	14
ES	20	21	17	21	13	18	7	6	123	15
FI	18	14	11	12	26	25	10	7	123	16
IL	11	26	19	23	18	30	4	5	136	17
NZ	29	24	15	13	14	13	17	13	138	18
PT	28	29	22	20	25	6	12	12	154	19
TW	26	12	20	14	28	32	8	14	154	20
DE	22	25	23	22	12	20	21	23	168	21
AT	10	23	26	26	17	15	28	26	171	22
KR	27	19	16	17	21	29	20	22	171	23
ZA	2	28	24	29	24	14	27	28	176	24
PL	3	22	33	31	27	4	30	29	179	25
IN	6	18	28	28	29	10	32	32	183	26
FR	7	11	31	30	19	26	31	31	186	27
BR	30	31	27	24	23	2	26	25	188	28
JP	25	15	25	19	22	31	29	27	193	29
TR	32	32	29	27	31	27	14	9	201	30
IR	31	27	30	25	32	22	24	21	212	31
MX	17	30	32	32	30	17	25	30	213	32

**Table 6.** Impact parameters (left table) for PCR papers, 2013–22, in 19 research domains and ranking of the domains (right table).

	JIF	ACI	DL	WS			JIF	ACI	DL	WS	Sum	Rank
ADVA	3.35	12.7	1.23	234		ADVA	11	1	5	1	18	1
AIDS	3.41	6.45	0.66	0		ONCOL	5	5	9	5	24	2
CARDI	3.57	8.98	0.65	96		QUAL	12	2	8	3	25	3
COST	3.85	8.56	0.78	165		COST	3	10	11	2	26	4
COVID	3.72		1.28			RENAL	2	3	14	7	26	5
DIABE	4.12	9.20	0.77	44		INDI	10	4	10	8	32	6
MEDI	3.85	8.32	0.58	137		FAMI	16	7	1	11	35	7
EDUC	2.42	7.79	1.10	40		DIABE	1	6	12	16	35	8
ETHI	2.29	5.28	1.40	56		MEDI	4	11	17	4	36	9
FAMI	2.96	9.06	1.44	81		MENTA	14	13	2	10	39	10
INDI	3.36	10.1	0.84	117		COVID	7	10	4	19	40	11
MENTA	3.09	7.86	1.43	92		PAIN	13	9	13	6	41	12
ONCOL	3.74	9.55	0.91	133		CARDI	8	8	16	9	41	13
PAED	3.01	7.88	1.10	65		PAED	15	12	6	13	46	14
PAIN	3.15	8.67	0.74	123		RADI	6	15	19	12	52	15
QUAL	3.22	10.4	1.03	145		ETHI	19	18	3	14	54	16
RADI	3.74	7.56	0.38	72		EDUC	18	14	7	17	56	17
RENAL	3.9	10.2	0.70	118		AIDS	9	17	15	18	59	18
SURG	2.91	7.21	0.43	50		SURG	17	16	18	15	66	19
